# Association of short term exposure to Asian dust with increased blood pressure

**DOI:** 10.1038/s41598-020-74713-6

**Published:** 2020-10-19

**Authors:** Masanobu Ishii, Tomotsugu Seki, Kenji Sakamoto, Koichi Kaikita, Yoshihiro Miyamoto, Kenichi Tsujita, Izuru Masuda, Koji Kawakami

**Affiliations:** 1 Graduate School of Medical Sciences, Kumamoto University, Kumamoto, Japan; 2 Department of Pharmacoepidemiology, Graduate School of Medical and Public Health, Kyoto University, Yoshida, Konoe-cho, Sakyo-ku, Kyoto, 606-8501 Japan; 3 National Cerebral and Cardiovascular Center, Suita, Japan; 4 Takeda Hospital Group, Kyoto, Japan

**Keywords:** Natural hazards, Cardiology, Risk factors

## Abstract

Air pollution causes hypertension, cardiovascular disease, and mortality. Asian dust (AD) reportedly induces asthma or acute myocardial infarction along with air pollution, but its impact on blood pressure (BP) is unknown. We investigated the association between short-term AD exposure and BP fluctuations in 300,952 individuals whose BP was measured during April 2005–March 2015 and divided them into AD and non-AD groups based on visitation for AD-related events. AD’s occurrence, air pollutants’ concentration (suspended particulate matter, SO_2_, NO_2_, photochemical oxidants), and meteorological variables (mean ambient temperature, relative humidity) were obtained from a monitoring station; AD events correlated with decreased visibility (< 10 km). We observed 61 AD days, with 3897 participants undergoing medical check-ups. Short-term AD exposure at lag day-0 was significantly associated with higher systolic BP (SBP), diastolic BP (DBP), and pulse rate (PR) risk (β = 1.85, 95% confidence interval (CI) 1.35–2.35 for SBP, β = 2.24, 95% CI 1.88–2.61 for DBP, β = 0.52, 95% CI 0.14–0.91 for PR) using multi-pollutant model. Population-attributable fractions exposed to AD were 11.5% for those with elevated SBP (SBP ≥ 120 mmHg) and 23.7% for those with hypertension (SBP ≥ 140 mmHg or DBP ≥ 90 mmHg). This study showed a strong association between short-term AD exposure and increased SBP and DBP.

## Introduction

Hypertension is both a medical and a social problem that needs to be solved, both due to a large disease^1,2^ and economic burden, as well as to an increasing number of patients^3^. Therefore, additional intensive pharmacological therapy for hypertension is strongly recommended as a cost-effective solution^4,5^. To prevent hypertension, a risk factor-intervention, especially lifestyle-related is imperative^6,7^. Adding to the known risk factors, air pollution has been recently recognized as an environmental risk factor for hypertension^8,9^. Intervening against this risk factor may reduce disease prevalence and alleviate the economic burden.

Asian dust (AD) is a part of desert dust whose sandy particulate matter originated in the Central Asian and Chinese desert regions; it is known to be hazardous even in regions far from the desert and its effects include ischemic heart disease^10–12^. During westerly transportation, AD’s capability of attachment on a micro-organism or other harmful substances—such as nitrogen and sulfur oxides produced by factories using fossil fuels and automobile factories—is area dependent (ranging from industrial, urban, or rural areas)^13,14^. Previous animal studies showed that AD’s particulate matter is correlated with oxidative stress and systemic inflammation, resulting in a mean BP and heart rate increase^15–17^; however, no reports have shown an association between AD and increased blood pressure in humans. Should AD exposure be associated with elevated BP in humans, avoiding AD would reduce hypertension-related disease prevalence.

We conducted a cross-sectional study based on health check-up data to estimate whether short-term AD exposure would adversely affect BP in humans in an attempt to address the lack of current knowledge highly relevant to our pending questions in the field of preventive and environmental cardiology.

## Methods

### Study design, participants, and data collection

This was a retrospective cross-sectional study. We included the data of 325,017 participants who underwent a comprehensive medical check-up and BP measurement at the Takeda Hospital Group between April 2005 and March 2015, of whom 24,065 were excluded as they were aged under 20 years (n = 1496) and were treated with antihypertensive agents (n = 22,569); 300,952 participants were finally included in the analysis (Supplementary Fig. S1).

Participant characteristics were obtained by an administered questionnaire, and their data included those on age, sex, smoking status (current or not), medical history, and medication. Height, weight, BP, and pulse rate were measured as previously described^18^. Briefly, after exposure to outside air, the BP of the participants of this study was measured within 2 h. Following more than 5 min of seated rest, we measured the brachial BP twice with a more than 1-min interval while an average of 2 BP measurements was used for analysis in this study. Simultaneous BP measurements were performed by trained nurses if necessary. Body Mass Index (BMI) was calculated as weight (kg) divided by height squared (m^2^), as previously described^18^.

### Ethical approval

This study was performed under the Declaration of Helsinki and its amendments and was approved by the ethics committees at Kyoto University, Kumamoto University, and Takeda Hospital Group. This study was exempted from the requirement for individual informed consent because all data were anonymized. The participants were notified regarding the use and analysis of their data through the institutes’ homepage or posters and were able to opt-out upon request (opt-out method).

### AD, air pollutants, and climate parameters

AD day was defined as a day when AD events resulted in visibility decrease (< 10 km) on ground level measured as previously described by local manned-meteorological observatories^10–12^. The participants were divided into 2 groups: those who visited on AD day (AD group) or non-AD day (non-AD group). Data for AD days and climate parameters such as the daily mean ambient temperature and relative humidity measured at Kyoto Local Meteorological Observatory (distance to the health check-up center: 3.73 km) were obtained from the databank published by the Japan Meteorological Agency (https://www.data.jma.go.jp/gmd/env/kosahp/kosa_data_index.html, and https://www.data.jma.go.jp/gmd/risk/obsdl/index.php). Data regarding the daily ambient concentration of other air pollutants (suspended particulate matter: SPM; sulfur dioxide: SO_2_; nitrogen dioxide: NO_2_; photochemical oxidants: Ox), which were measured at the Kyoto City Sanitation and Environment Institute (distance to the health check-up center: 3.2 km), were obtained from the *Kankyosuchi* database (in Japanese) published by the National Institute for Environmental Studies (https://www.nies.go.jp/igreen/index.html). The environmental measurement on the day of the visit was used as the exposure.

### Outcomes definition

We found that SBP and diastolic BP (DBP) were affected by AD. BP categories were: elevated SBP that was defined as SBP ≥ 120 mmHg, high BP (Stage 1 of hypertension) that was defined as SBP ≥ 130 mmHg or DBP ≥ 80 mmHg, and hypertension (Stage 2 of hypertension) that was defined as SBP ≥ 140 mmHg or DBP ≥ 90 mmHg, according to the current guideline^19^. In addition, we evaluated the association between AD exposure and pulse rate (PR).

### Statistical analysis

The partial regression coefficient (β) and 95% confidence interval (CI) of the effect of AD on those outcomes in the participants were calculated using a generalized linear model, to estimate the association between short-term exposure to AD and SBP, DBP, and PR. This study used a single-lag day-0 according to the analysis result of the single- and cumulative-day lag effects of AD (Supplementary Fig. S2).

Moreover, we adjusted differences in baseline characteristics and meteorological exposures of the participants with propensity score matching to reduce the effect of known possible confounders. The predicted probability of visiting on an AD day was calculated by applying a logistic regression model, using all clinically and climatically relevant variables such as age, sex, mean ambient temperature (day-0), and relative humidity (day-0). One AD group participant was matched with 1 participant in the non-AD group using nearest-neighbor matching within a caliper width of 0.01 without replacement. A comparison of the baseline characteristics between the AD and non-AD groups in the entire and matched population was performed using the standardized difference, whereby an absolute standardized difference above 0.10 represents meaningful imbalance.

Additionally, inverse probability of treatment weighting (IPTW) analysis using the same predicted probability used in the propensity score matching was performed as sensitivity analysis for the same outcomes, using a generalized estimating equation to confirm the results’ robustness. The relative risk (RR) and 95% CI of the categories of BP associated with AD exposure in the entire population were calculated using a Poisson regression model. In the multivariable analysis, the baseline characteristics, 24-h averages (day-0) of mean temperature and humidity, and 24-h mean concentrations (day-0) of SPM, SO_2_, NO_2_, or Ox were used as covariates. The effect estimates were calculated from Poisson regression and reported as RR in mmHg for blood pressure and in bpm for pulse rate for each 1-μg/m^3^ increase in 24-h mean concentrations of SPM, SO2, NO_2_, or Ox. The population-attributable fractions (PAFs) of the categories of BP associated with AD exposure were calculated from RRs of the multi-pollutant model including SPM as a covariate in the entire population and were defined as p × [(RR-1)/p × (RR-1) + 1], p indicating exposure prevalence, as previously described^20^. We performed subgroup analysis with baseline characteristics such as age, sex, BMI, smoking status, and presence of diabetes mellitus. The partial regression coefficient (β) and 95% CIs adjusted for the multi-pollutant model, including SPM, were calculated using a generalized linear model to estimate the association between AD exposure and SBP, DBP, or PR testing the interaction for each subgroup. Two-sided p < 0.05 was considered as statistically significant, and the statistical analyses were performed using SAS version 9.4 (SAS Institute, Cary, NC) and SPSS version 23.0 (IBM Corporation, Armonk, NY).

## Results

### Baseline characteristics of participants and environmental data

During the 10-year study period, we observed 61 AD days in the city of Kyoto in Japan. Among the 300,952 participants, 3897 visited on an AD day (AD group) and 297,055 visited on a non-AD day (non-AD group) (Supplementary Fig. S1). Table 1 shows the clinical and climatic characteristics of the participants. In the entire population, baseline characteristics were comparable between AD and the non-AD group, except for smoking habits and climate parameters. Following propensity score matching, both groups’ characteristic distribution was well-balanced; however, the absolute standardized difference of humidity was 0.12. The concentration of air pollutants, especially that of SPM, was significantly higher in the AD group than in the non-AD (Table 1).

**Table 1:** Clinical and climatic characteristics of entire and matched population.

	Entire population			Matched population		
	AD group n = 3897	Non-AD group n = 297,055	Standardized difference	AD group n = 3897	Non-AD group n = 3897	Standardized difference
Age, years, mean (SD)	42.4 (13.2)	43.2 (13.0)	0.06	42.4 (13.2)	42.4 (13.1)	0.00
20 ≤, ≤ 30, n (%)	900 (23.1)	60,796 (20.5)	0.06	900 (23.1)	880 (22.6)	0.01
31 ≤, ≤ 40, n (%)	928 (23.8)	72,752 (24.5)	0.02	928 (23.8)	986 (25.3)	0.03
41 ≤, ≤ 50, n (%)	906 (23.2)	71,702 (24.1)	0.02	906 (23.2)	884 (22.7)	0.01
51 ≤, ≤ 60, n (%)	778 (20.0)	60,555 (20.4)	0.01	778 (20.0)	768 (19.7)	0.01
61 ≤, n (%)	385 (9.9)	31,250 (10.5)	0.02	385 (9.9)	379 (9.7)	0.01
Male, n (%)	2,029 (52.1)	145,849 (49.1)	0.06	2029 (52.1)	1913 (49.1)	0.06
BMI, kg/m^2^, mean (SD)	22.2 (3.3)	22.1 (3.4)	0.01	22.2 (3.3)	22.1 (3.3)	0.03
Current smoking, n (%)	454 (11.6)	44,779 (15.1)	0.10	454 (11.6)	546 (14.0)	0.07
Heart disease, n (%)	33 (0.8)	2838 (1.0)	0.02	33 (0.8)	30 (0.8)	0.00
Renal disease, n (%)	2 (0.1)	212 (0.1)	0.00	2 (0.1)	5 (0.1)	0.00
Prior cerebral infarction, n (%)	9 (0.2)	938 (0.3)	0.02	9 (0.2)	16 (0.4)	0.04
**Medication**						
Lowering blood glucose, n (%)	30 (0.8)	2761 (0.9)	0.01	30 (0.8)	41 (1.1)	0.03
Anti-dyslipidemia agent, n (%)	83 (2.1)	7771 (2.6)	0.03	83 (2.1)	108 (2.8)	0.05
Systolic blood pressure, mmHg, mean (SD)	112.7 (16.2)	112.8 (16.6)	0.01	112.7 (16.2)	112.6 (16.1)	0.01
Diastolic blood pressure, mmHg, mean (SD)	69.5 (12.3)	70.0 (12.3)	0.04	69.5 (12.3)	69.8 (12.5)	0.02
Pulse rate, bpm, mean (SD)	75.1 (11.6)	73.7 (11.6)	0.12	75.1 (11.6)	73.9 (11.5)	0.10
**Climate**						
Mean temperature, ℃, mean (SD)	15.7 (4.9)	16.8 (8.8)	0.15	15.7 (4.9)	15.6 (8.2)	0.01
Humidity, %, mean (SD)	54.8 (10.3)	64.6 (9.8)	0.97	54.8 (10.3)	56.1 (11.2)	0.12
SPM, µg/m^3^, mean (SD)	49.1 (30.4)	19.0 (10.8)	1.32	49.1 (30.4)	20.1 (10.4)	1.28
SO_2_, ppb, mean (SD)	4.7 (1.5)	3.8 (1.3)	0.64	4.7 (1.5)	4.2 (1.4)	0.34
NO_2_, ppb, mean (SD)	17.2 (7.1)	14.4 (6.7)	0.41	17.2 (7.1)	14.2 (6.4)	0.44
Ox, ppb, mean (SD)	36.7 (14.2)	29.5 (12.7)	0.53	36.7 (14.2)	35.3 (14.0)	0.10

Values are mean (SD) or n (%).

*AD* Asian dust, *SD* standard deviation, *BMI* Body Mass Index, *SPM* suspended particulate matter, *NO*_*2*_ nitrogen dioxide, *SO*_*2*_ sulfur dioxide, *Ox* photochemical oxidants.

### Main analysis

Short-term exposure to AD was significantly associated with an increased risk of higher SBP, DBP, and PR [β = 1.85, 95% CI 1.35–2.35 for SBP, β = 2.24, 95% CI 1.88–2.61 for DBP, β = 0.52, 95% CI 0.14–0.91 for PR, respectively] in a multi-pollutant model adjusted for SPM (Table 2). Moreover, AD exposure was significantly associated with increases in SBP, DBP, and PR in models adjusted for gaseous air pollutants such as SO_2_, NO_2_, or Ox, in addition to SPM. These significant associations were generally observed in propensity score matching of the multi-pollutant models; however, some were not statistically significant in DBP (Table 2).

**Table 2:** Effect of Asian dust on systolic blood pressure, diastolic blood pressure, and pulse rate.

Multi-pollutant model	Systolic blood pressure				Diastolic blood pressure				Pulse rate			
	β	95% CI	SE	p value	β	95% CI	SE	p value	β	95% CI	SE	p value
**Entire population**^a^												
+ SPM	1.85	1.35, 2.35	0.253	< 0.001	2.24	1.88, 2.61	0.188	< 0.001	0.52	0.14, 0.91	0.195	0.007
+ SPM, SO_2_	2.33	1.82, 2.84	0.258	< 0.001	2.87	2.49, 3.24	0.192	< 0.001	0.60	0.21, 0.99	0.199	0.003
+ SPM, NO_2_	1.60	1.10, 2.10	0.255	< 0.001	1.87	1.50, 2.24	0.189	< 0.001	0.68	0.30, 1.07	0.197	0.001
+ SPM, Ox	1.91	1.41, 2.41	0.253	< 0.001	2.32	1.95, 2.68	0.188	< 0.001	0.52	0.14, 0.90	0.195	0.008
**Matched population**^b^												
+ SPM	1.10	0.25, 1.96	0.436	0.011	0.46	− 0.20, 1.11	0.335	0.16	1.17	0.56, 1.77	0.311	< 0.001
+ SPM, SO_2_	1.05	0.20, 1.91	0.438	0.016	0.43	− 0.23, 1.09	0.337	0.20	1.16	0.54, 1.77	0.312	< 0.001
+ SPM, NO_2_	1.22	0.35, 2.08	0.442	0.006	0.72	0.06, 1.39	0.339	0.033	0.99	0.38, 1.61	0.315	0.002
+ SPM, Ox	1.09	0.23, 1.94	0.436	0.013	0.47	− 0.19, 1.13	0.335	0.16	1.16	0.55, 1.77	0.311	< 0.001
**IPTW (sensitivity analysis)**												
+ SPM	2.75	1.49, 4.01	0.642	< 0.001	2.24	1.27, 3.20	0.491	< 0.001	1.47	0.58, 2.36	0.454	0.001
+ SPM, SO_2_	2.53	1.30, 3.75	0.626	< 0.001	2.04	1.10, 2.97	0.477	< 0.001	1.42	0.54, 2.29	0.445	0.001
+ SPM, NO_2_	2.89	1.62, 4.16	0.648	< 0.001	2.51	1.54, 3.48	0.495	< 0.001	1.22	0.33, 2.12	0.458	0.008
+ SPM, Ox	2.67	1.44, 3.91	0.629	< 0.001	2.18	1.24, 3.12	0.481	< 0.001	1.43	0.56, 2.30	0.445	0.001

*CI* confidence interval, *SE* standard error, *SPM* suspended particulate matter, *NO*_*2*_ nitrogen dioxide, *SO*_*2*_ sulfur dioxide, *Ox* photochemical oxidants, *IPTW* inverse probability of treatment weighting.

^a^Adjusted for sex, age category, BMI category, smoking status, mean temperature, and relative humidity.

^b^Adjusted for relative humidity.

**Figure 1:**
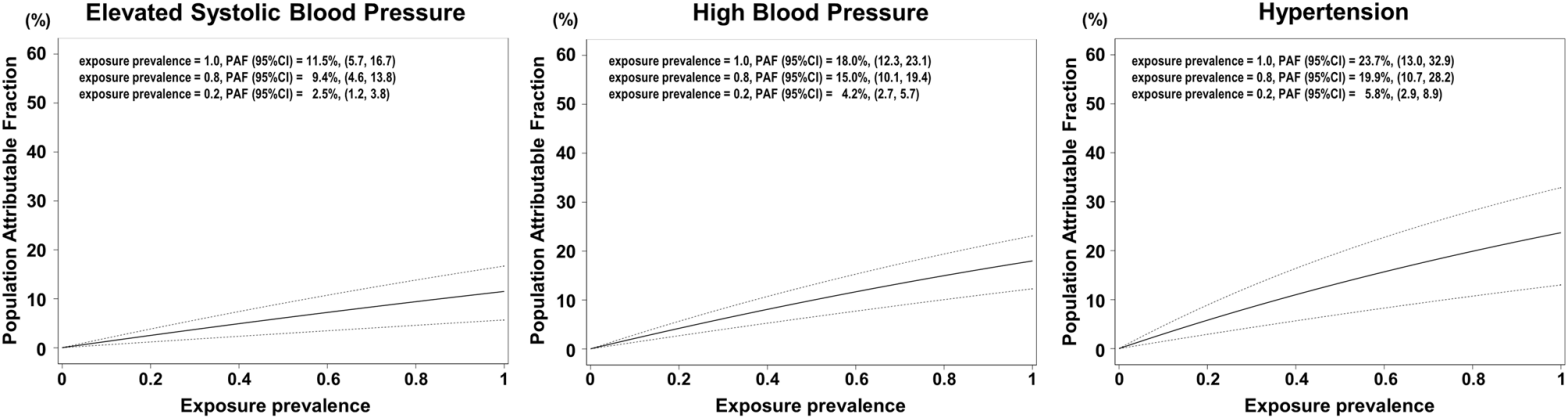
Population attributable fractions of the categories of blood pressure associated with short-term exposure to Asian dust. These line and dot graphs show population attributable fractions and the 95% confidence intervals for the elevated systolic blood pressure (left panel), high blood pressure (middle panel), and hypertension (right panel). *PAF* population attributable fraction, *CI* confidence interval.

AD short-term exposure was associated with elevated SBP (adjusted RR; 1.13, 95% CI 1.06–1.20), high BP (adjusted RR; 1.22, 95% CI 1.14–1.30), and hypertension (adjusted RR; 1.31, 95% CI 1.15–1.49) (Table 3). Figure 1 shows the PAFs of the categories of BP attributable to AD exposure with a penalized spline. Since the day when AD events occurred, participants are considered exposed to AD before the health check-up assuming exposure prevalence at 100%. Based on this assumption, PAF of hypertension attributable to AD exposure was 23.7% (95% CI 13.0–32.9) (Fig. 1, right panel), that of elevated SBP was 11.5% (95% CI 5.7–16.7) (Fig. 1, left panel), and that of high BP was 18.0% (95% CI 12.3–23.1) (Fig. 1, middle panel).

**Table 3:** Relative risk of short-term exposure to Asian dust on the categories of blood pressure in the entire population.

Multi-pollutant model	Elevated systolic blood pressure (SBP ≥ 120 mmHg)			High blood pressure (SBP ≥ 130 mmHg or DBP ≥ 80 mmHg)			Hypertension (SBP ≥ 140 mmHg or DBP ≥ 90 mmHg)		
	RR	95% CI	p value	RR	95% CI	p value	RR	95% CI	p value
+ SPM	1.13	1.06, 1.20	< 0.001	1.22	1.14, 1.30	< 0.001	1.31	1.15, 1.49	< 0.001
+ SPM, SO_2_	1.16	1.09, 1.23	< 0.001	1.27	1.18, 1.36	< 0.001	1.43	1.25, 1.62	< 0.001
+ SPM, NO_2_	1.12	1.05, 1.19	< 0.001	1.19	1.11, 1.27	< 0.001	1.26	1.11, 1.43	< 0.001
+ SPM, Ox	1.14	1.07, 1.21	< 0.001	1.22	1.14, 1.31	< 0.001	1.32	1.17, 1.51	< 0.001

Models were adjusted for sex, age category, BMI category, smoking status, mean temperature, and relative humidity.

*SPM* suspended particulate matter, *NO*_*2*_ nitrogen dioxide, *SO*_*2*_ sulfur dioxide, *Ox* photochemical oxidants.

### Sensitivity analysis

We performed a sensitivity analysis to assess the robustness of the association between AD exposure and outcomes (Table 2). Regarding the results of IPTW in the multi-pollutant model, the short-term exposure to AD was significantly associated with higher SBP, DBP, and PR, thus being consistent with the results of the main analysis.

### Subgroup analysis

**Figure 2:**
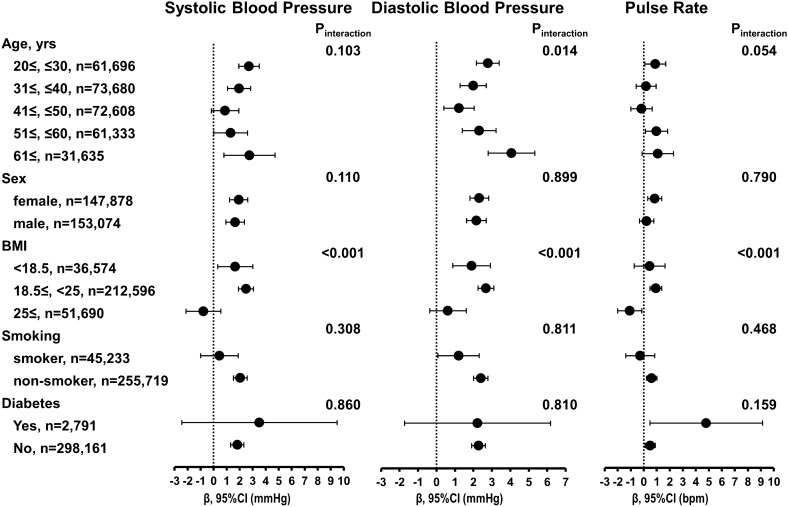
Association of outcomes with short-term exposure to Asian dust in subgroup. The partial regression coefficient (β) and 95% confidence interval were adjusted for baseline characteristics, ambient mean temperature, relative humidity, and concentration of suspended particulate matter. *BMI* Body Mass Index.

Although aging is an important factor in BP, SBP and DBP were inversely associated with PR (Supplementary Fig. S3). Subgroup analysis in the entire population showed that the significant interaction between AD exposure and the categories of BMI was observed in SBP, DBP, and PR (Fig. 2). The borderline effect of interaction between AD exposure and age was observed in SBP and PR, despite being significant in DBP.

## Discussion

This large-scale cross-sectional study of a healthy Japanese population showed that short-term exposure to AD was significantly associated with an increase in SBP, DBP, and PR, while AD exposure-related PAF of hypertension was 23.7%, indicating that avoiding AD exposure could prevent the development of hypertension in about one-quarter of the patients. Furthermore, the result of the subgroup analysis provided evidence that age and BMI were effect modifiers in the increasing BP associated with AD exposure. To the best of our knowledge, this is the first report to clarify the potential effects of AD on blood pressure in healthy individuals.

Environmental factors, especially in air pollution, have been recognized as novel hypertension risk factors^8,9^. Although no previous studies indicated any association between AD exposure and BP fluctuations, associations between particulate matter and BP have been reported. A previous population-based cohort study showed that long-term exposure to particulate matter (≤ 2.5 µm in aerodynamic diameter) (PM_2.5_) was associated with the development of hypertension (hazard ratio; 1.13, 95% CI 1.05–1.22, per 10-µg/m^3^ increase of PM_2.5_) in 35,303 non-hypertensive adults^21^. Another study showed that short-term exposure to ambient PM_2.5_ was associated with increases in both systolic and diastolic BP levels in healthy normotensive participants, especially in overweight adults (BMI ≥ 25 kg/m^2^)^22^. Additionally, exposure to air pollutants other than PM_2.5_, such as PM_10_, NO_2_, SO_2_, and O_3,_ was associated with increases in SBP in patients with type-2 diabetes mellitus^23^. In addition to these observations, the effect of air pollution on BP was confirmed in previous controlled trials^24^. Cosselman et al. investigated the traffic-related air pollution exposure’s effect on the rapid reaction of BP^24^. The crossover exposure study in 45 young non-smoker adults showed that SBP increased rapidly and peaked 1 h following the commencement of diesel exhaust exposure without statistically significant changes in DBP and HR^24^. Consistent with that result, the participants of this study were also exposed within 2 h prior to BP measurement; thus, the result of this study might suggest an acute effect of short-term exposure to AD on BP in humans.

Since the health check-up center of the Takeda Hospital Group faces the main road that has heavy traffic, locally generated air pollutants such as harmful exhaust from automobiles might have affected the BP of participants of this study. To reduce the effect of other air pollutants as a confounder, the multivariable model was employed with adjustment for SPM, NO_2_, SO_2_, and Ox. Following adjustment, short-term exposure to AD was the independent risk factor of high BP. AD constitutes sandy particulate matter originating in the deserts of Mongolia and Kazakhstan, and the size of AD found in Japan ranges from 0.5 to 5 µm, with a peak at 4 µm^25^. During airborne eastward transportation, AD carries various harmful substances, such as other air pollutants and microorganisms^13,14^. Previous studies also suggested that both the components of AD and AD-induced inflammatory response varied depending on the source of particulate matter and passage routes^26–28^. Based on this evidence, we speculated that—although the particulate matter of AD itself caused little harm—the AD-attached matrix during transportation might present an adverse health effect. To maintain high air quality, global cooperation—rather than only local/regional effort—is needed to control air pollution.

Our subgroup analysis (Fig. 2) showed significant interaction in the BMI category in the association between short-term exposure to AD and BP. The point estimate was higher in the lower BMI (BMI < 18.5, 18.5 to 25) than in the higher BMI (BMI ≤ 25) group; in addition, non-smokers had a larger BP increase than smokers, though, the interaction was non-significant. This might be due to healthier participants’ lower exposure to cardiometabolic risk factors such as aging, obesity, and smoking, thus concluding that AD might have a greater impact on them. However, regarding the age category, BP significantly increased both in the younger and the elderly as we confirmed the same as the above- or larger- intake of dusty air due to their high outdoor activity. Conversely, the elderly seemed to have less intake of ambient air due to their low activity, thus proposing a separate explanation. There is no supporting evidence that the elderly were susceptible to AD-induced BP elevation. Therefore, further investigation is needed to elucidate the mechanism responsible for the difference in the effects of AD exposure due to age differences.

### Limitations

This study has several limitations. First, regarding the nature of the observational study, the causal relationship between AD exposure and fluctuated BP in humans could not be confirmed. However, a clinical trial randomly assigning AD exposure cannot be ethically conducted. An interventional trial preventing AD exposure might be possible in examining the effect of AD exposure on BP in the future. Second, misclassification of AD exposure might have affected the result since grouped representative data measured by the nearest monitoring station were used as exposure instead of individual exposure data thus having the result of this study potentially underestimated by this non-differential misclassification. Third, PM_2.5_ is one of the major particulate air pollutants in the world; however, it could not be adjusted because it had not been measured at the local monitoring site prior to October 2011. Moreover, no data for PM_2.5_ was available from the site. Fourth, unmeasured time-varying variables may affect the findings of this study because of the long study periods. For instance, Adar S et al. demonstrated a longitudinal study of investigating the impact of air pollution on blood pressure^29^. Although significant associations between higher concentrations of air pollutants and higher blood pressure were observed in models adjusted for all risk factors, including time-varying age but not calendar time, no associations were observed after additional adjustment for calendar time^29^. Fifth, this study could not investigate the association between AD exposure and BP increase in patients with hypertension because the effect of antihypertensive medication might be biased in the investigation of that association. However, the effect of air pollution on BP is a clinically important issue for patients with hypertension. Future prospective investigation to reduce the bias of management of BP control with antihypertensive agents is needed in the population. Sixth, personal protection equipment (PPE), such as masks, is useful for preventing AD exposure. Therefore, the AD-BP association might be underestimated in participants wearing PPE. Unfortunately, we did not investigate whether the participants were wearing their PPE before the health check-ups. Therefore, we could not evaluate PPE’s effect on the association between AD exposure and BP fluctuations.

### Conclusions

This study showed that short-term exposure to AD was significantly associated with an increase in SBP, DBP, and PR, almost consistently with the results of propensity score matching and weighting analysis. Additionally, the subgroup analysis showed that age and BMI were effect modifiers in the increasing BP associated with AD exposure. Since the PAF of hypertension attributable to AD exposure was relatively high, avoiding exposure to AD may prevent the development of hypertension in healthy adults.

## Supplementary information

Supplementary Information.
