# Supplementary material for: Association of short term exposure to Asian dust with increased blood pressure
Source: Sci Rep. 2020 Oct 19;10:17630. doi: 10.1038/s41598-020-74713-6 (PMC7572380; doi:10.1038/s41598-020-74713-6)
Supplement: Supplementary file 1 — Supplementary Information. [file 41598_2020_74713_MOESM1_ESM.docx]

**Supplementary Materials, Association of Short term Exposure to Asian Dust with Increased Blood Pressure**

Masanobu Ishii, MD, MPH, PhD^1^; Tomotsugu Seki, MD, MPHT^2^; Kenji Sakamoto, MD, PhD^1^; Koichi Kaikita, MD, PhD^1^; Yoshihiro Miyamoto, MD, PhD^3^; Kenichi Tsujita, MD, PhD^1^; Izuru Masuda, MD, PhD^4^; Koji Kawakami, MD, PhD^2^

^1^Graduate School of Medical Sciences, Kumamoto University, Kumamoto, Japan

^2^Graduate School of Medicine and Public Health, Kyoto University, Kyoto, Japan

^3^National Cerebral and Cardiovascular Center, Suita, Japan

^4^Takeda Hospital Group, Kyoto, Japan

**TABLE OF CONTENTS**

**Supplementary figure 1**: Study Flow Chart………………….…………………….………..…p2

**Supplementary figure 2**: The Single- and Cumulative-day Lag Effects of Asian Dust on Systolic Blood Pressure, Diastolic Blood Pressure, and Pulse Rate…………………………………..…p3

**Supplementary figure 3**: Comparison of Systolic Blood Pressure, Diastolic Blood Pressure, and Pulse Rate Between Asian Dust and non-Asian Dust Group Stratified by Age………………...p5

**
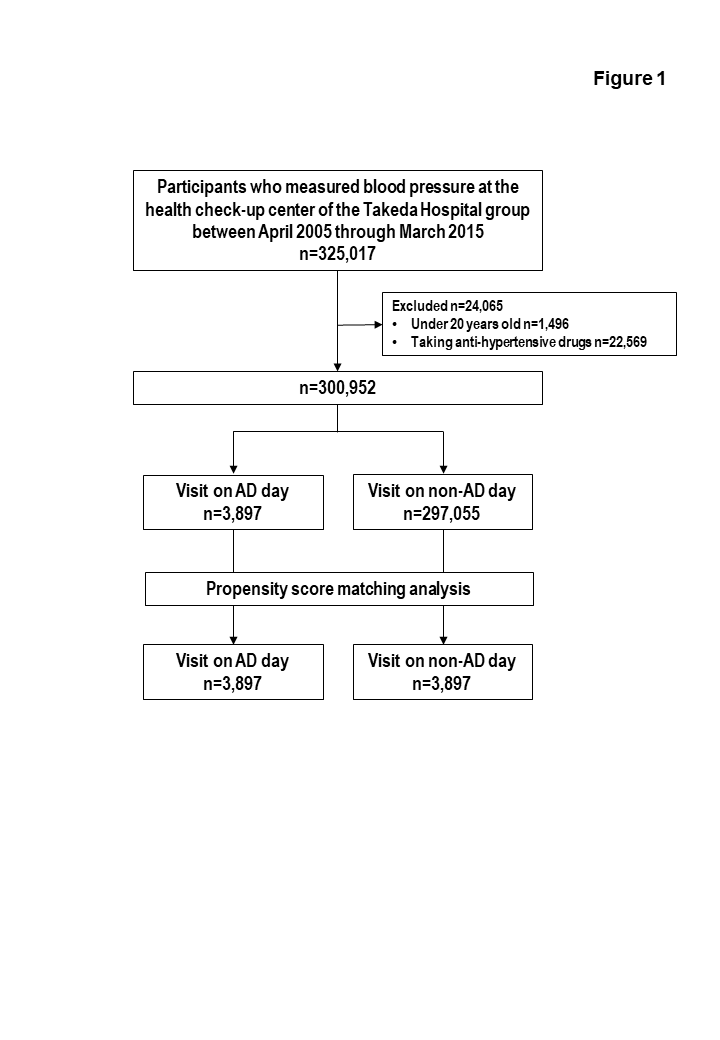
Supplementary figure 1. Study Flow Chart**

This chart shows enrollment criteria and flow of participants as they were grouped by whether or not Asian dust occurred on the day of the visit. AD indicates Asian dust.

**
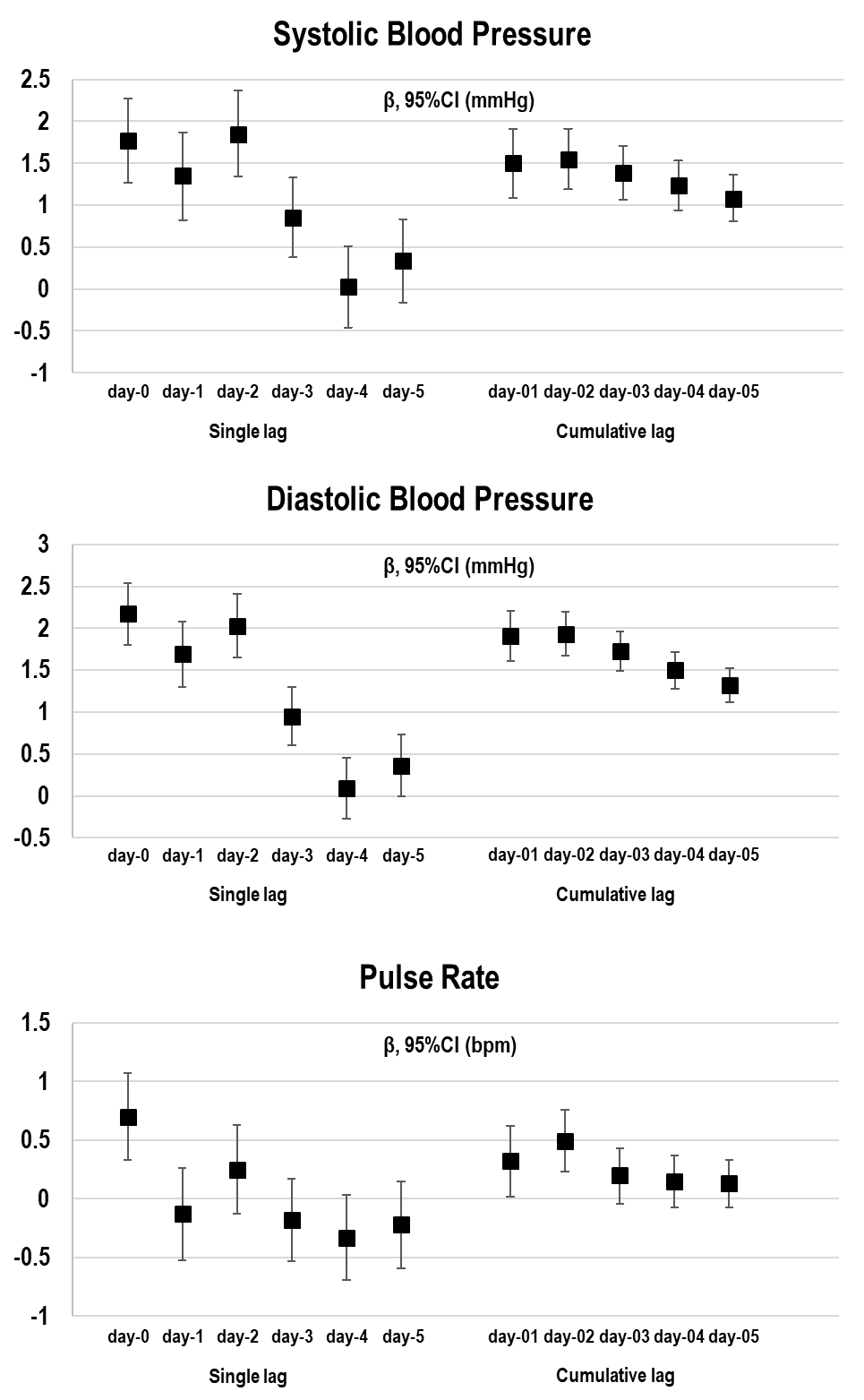
**

**Supplementary figure 2. The Single- and Cumulative-day Lag Effects of Asian Dust on Systolic Blood Pressure, Diastolic Blood Pressure, and Pulse Rate**

This figure shows the β and 95% CI of the single and cumulative lag effect of the short-term exposure to Asian dust using a generalized liner model adjusted for age, sex, body mass index, smoking habit, mean temperature (day-0 to day-2), relative humidity (day-0 to day-2), and suspended particulate matter (day-0 to day-2), indicating a greater impact of Asian dust exposure on single lag day-0 on systolic blood pressure, diastolic blood pressure, and pulse rate.

**
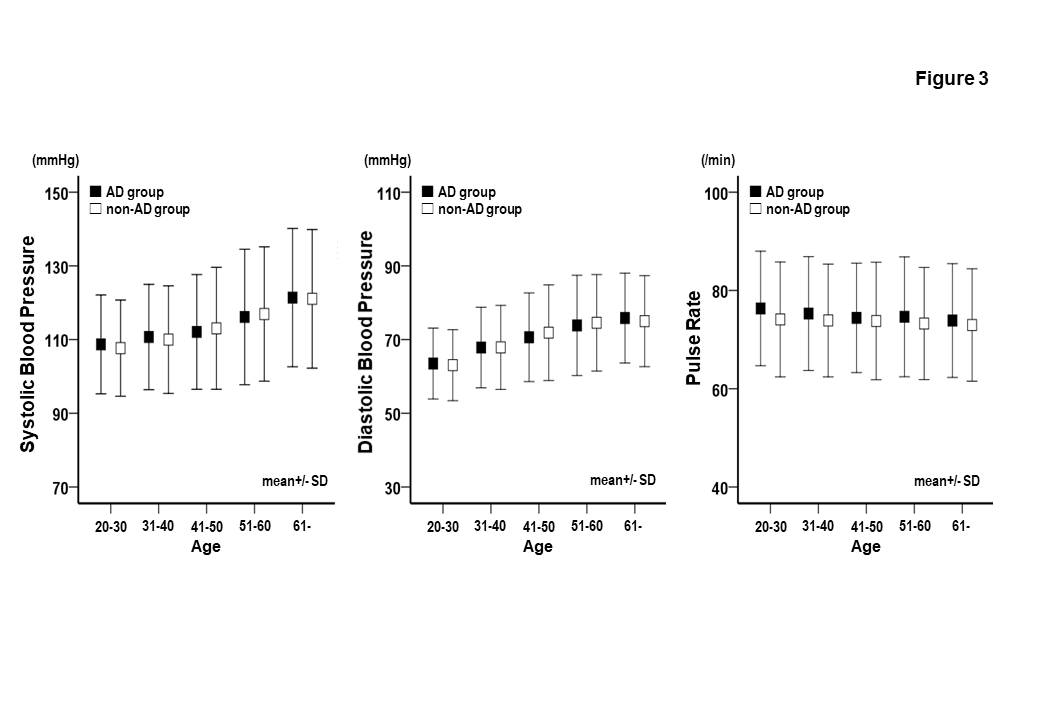
**

**Supplementary figure 3.** **Comparison of Systolic Blood Pressure, Diastolic Blood Pressure, and Pulse Rate Between Asian Dust and non-Asian Dust Group Stratified by Age**

These plots and error bars show means and standard deviations of age-stratified systolic blood pressure, diastolic blood pressure, and pulse rate in participants who visited the health check-up center on Asian dust day and non-Asian dust day. AD indicates Asian dust, SD; standard deviation.
